# Graz Critical Limb Ischemia Score: A Risk Score for Critical Limb Ischemia in Peripheral Arterial Occlusive Disease

**DOI:** 10.1097/MD.0000000000001054

**Published:** 2015-07-13

**Authors:** Thomas Gary, Klara Belaj, Franz Hafner, Philipp Eller, Peter Rief, Gerald Hackl, Marianne Brodmann

**Affiliations:** From the Division of Angiology, Department of Internal Medicine, Medical University Graz, Graz, Austria.

## Abstract

Critical limb ischemia (CLI), a frequently encountered disorder, is associated with a high rate of limb amputation and mortality. To identify patients at high risk for CLI, we developed a simple risk score for peripheral arterial occlusive disease (PAOD).

In our cross-sectional study, we first evaluated 1000 consecutive PAOD patients treated at our institution from 2005 to 2007, documenting clinical symptoms, comorbidities, and concomitant medication. We calculated odds ratios (OR) in a binary logistic regression model to find possible risk factors for CLI. We then verified the score in a second step that included the 1124 PAOD patients we treated between 2007 and 2011.

In the first patient group, the greatest risk factors for CLI were age ≥75 years (OR 2.0), type 2 diabetes (OR 3.1), prior myocardial infarction (OR 2.5), and therapy with low molecular weight heparins (2.8). We scored 1 point for each of those conditions. One point was given for age between 65 and 75 years (OR 1.6) as well as for therapy with cardiac glycosides (OR 1.9) or loop diuretic therapy (OR 1.5). As statin therapy was protective for CLI with an OR of 0.5, we subtracted 1 point for those patients.

In the second group, we could prove that frequency of CLI was significantly higher in patients with a high CLI score. The score correlated well with inflammatory parameters (c-reactive protein and fibrinogen). We were also able to define 3 different risk groups for low (score −1 to 1), intermediate (score 2–4), and high CLI risk (score >4).

We developed a simple risk stratification scheme that is based on conditions that can be easily assessed from the medical history, without any laboratory parameters. This score should help to identify PAOD patients at high risk for CLI.

## INTRODUCTION

Though many patients suffer from peripheral arterial occlusive disease (PAOD), diagnosis is often delayed.^1^ If PAOD is not diagnosed and treated immediately, it is highly probable that the disease will progress to critical limb ischemia (CLI).^2^ This is an entity with high mortality and high risk of limb amputation. Although treatment options, especially endovascular treatment modalities, have improved in recent decades, mortality and amputation rates remain high.^3,4^

Various scoring systems have been established to identify patients at high risk for cardiovascular disease. The most renowned is the Framingham risk score, which distinguishes individuals at low, moderate, and high risk for coronary heart disease.^5^ This score includes clinical (eg, blood pressure) and laboratory parameters (eg, high density lipoprotein cholesterol).

Recently, the PROCAM score was published to evaluate the 10-year myocardial infarction (MI) risk to identify patients who might profit from more intense prevention and cardiovascular risk factor management.^6^ This score includes laboratory parameters (eg, cholesterol), clinical parameters (eg, blood pressure), and concomitant medication (antihypertensives). Although CLI is a common disorder, there is still no risk score to evaluate the CLI risk in PAOD patients. We therefore evaluated CLI risk factors in our PAOD cohort and developed a risk score, the Graz CLI score, which is easy to use with PAOD patients.

## METHODS

For our cross-sectional study, we formed a first cohort of 1000 patients with PAOD treated at our department between 2003 and 2007 to identify CLI risk factors and to develop our scoring model. We validated our scoring system with a second cohort, consisting of 1124 PAOD patients we treated between 2007 and 2011.

The inclusion criterion for our study was treatment at our institution for PAOD during the given time period; there were no exclusion criteria. The study was approved by the Institutional Review Board of the Medical University of Graz, Austria (IRB Number 24-506 ex 11/12). As this was a retrospective analysis of blinded data, the Board agreed that neither written nor verbal consent would be required.

PAOD was diagnosed and graded in our outpatient clinic on the basis of clinical evaluation, ankle brachial index, and duplex scan according to the TASC II criteria. Successive PAOD patients presenting at our outpatient angiology clinic were scheduled for admission to our ward for further treatment of their atherosclerotic disease. PAOD was graded according to the Fontaine classification. CLI was defined as PAOD presenting with ischemic rest pain and/or skin ulceration/gangrene in accordance with current guidelines and corresponding to Fontaine classes 3 and 4.^7^ Upon hospital admission, the patient's medical records were analyzed systematically for cardiovascular risk factors, comorbidities, and medication. Clinical symptoms were evaluated with a physical examination. Fasting blood samples were taken for laboratory studies.

### Statistical Analyses

The subjects’ clinical characteristics were analyzed with descriptive statistics. Groups were compared with the χ^2^ test for categorical values, the *t* test for normally distributed continuous variables, and the Mann–Whitney *U* test for nonnormally distributed continuous variables.

Univariate regression analysis was used to assess the influence of individual demographic data and comedication parameters on CLI. Variables exhibiting a significance level of *P* < 0.05 on univariate analysis were then entered into a multivariate binary logistic regression model. For all tests, the threshold *P* value for significance was *P* ≤ 0.05. SPSS 22.0 was used for all statistical analyses.

The validation cohort was divided into 3 risk groups according their CLI risk score. The occurrence of CLI was evaluated and a Jonckheere–Terpstra test performed to detect any statistical trend indicating that CLI rates increase with the CLI risk score.

## RESULTS

The patients’ characteristics are shown in Table 1. There were statistically significant differences between cohort 1 and cohort 2 with respect to the incidence of coronary artery disease (CAD; 326 [32.6%] vs 420 [37.4%], *P* = 0.03) and atrial fibrillation (149 [14.9%] vs 219 [19.4%], *P* = 0.01). With regard to concomitant medication we found differences in antiplatelet therapy (aspirin: 414 [41.4%] vs 379 [33.7%], *P* = 0.001) and clopidogrel (152 [15.2%] vs 287 [25.5%], *P* = 0.001). This might be because of the differences in the time periods when patients presented at our outpatient clinic, where cohort 1 was treated between 2004 and 2007 and cohort 2 was treated between 2007 and 2011. During the latter period, patients were increasingly treated with clopidogrel. Furthermore, CAD frequency differed significantly between groups also leading to a difference in concomitant medication (angiotensin receptor blocker and β-blocker: 112 [11.2%] vs 171 [15.2%], *P* = 0.01, and 381 [38.1%] vs 495 [44%], *P* = 0.01).

**TABLE 1:**
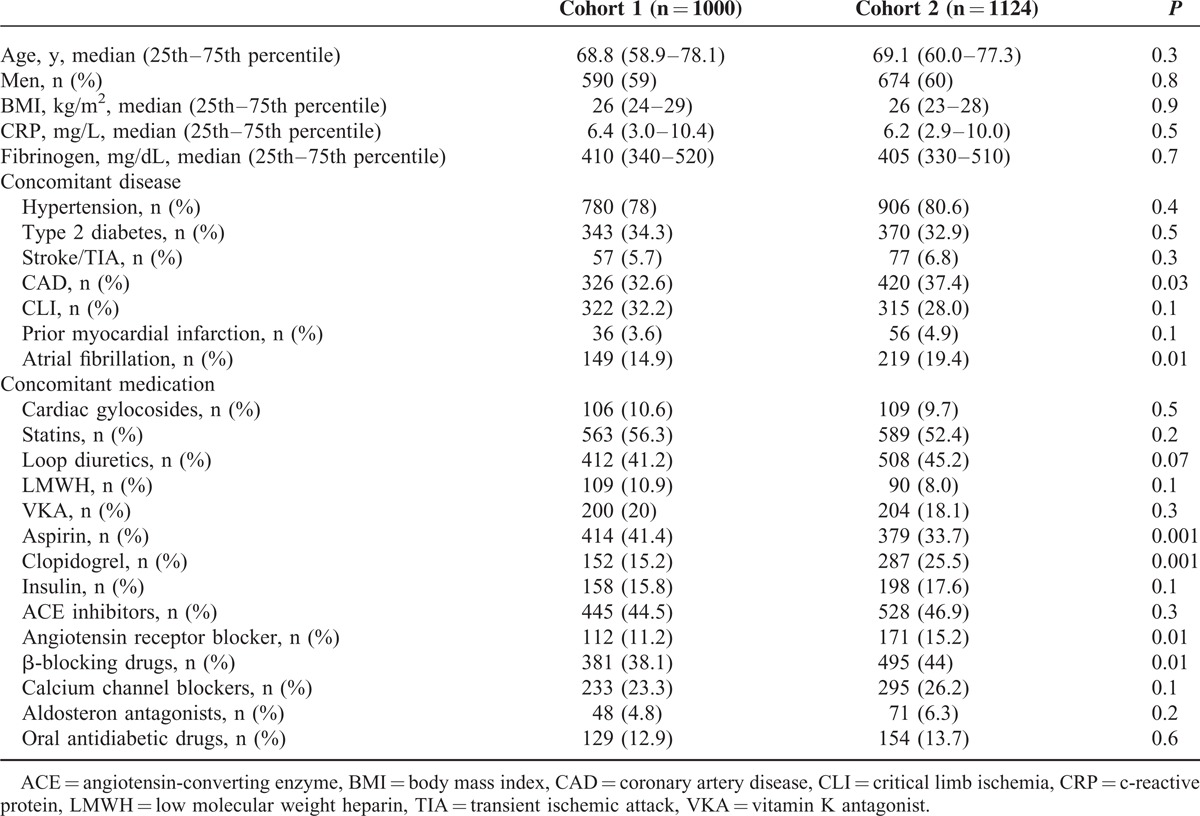
Patients’ Characteristics of Peripheral Arterial Occlusive Disease Patients Included in the Study (Cohort 1 and Cohort 2)

Parameters of medical history and concomitant medication were first evaluated in a univariate regression analysis for the occurrence of CLI in cohort 1 (Table 2). Variables exhibiting a significance level of *P* < 0.05 on univariate analysis were then entered into a multivariate binary logistic regression model. In cohort 1, the strongest risk factors for CLI in the multivariate regression model were age >75 years (OR 2.0 [95% confidence interval [CI] 1.3–3.0], *P* < 0.001), type 2 diabetes (OR 3.1 [95% CI 2.2–4.4], *P* < 0.001), prior MI (OR 2.5 [95% CI 1.1–5.5], *P* = 0.03), and therapy with low molecular weight heparin (LMWH) (2.8 [95% CI 1.7–4.4], *P* < 0.001). For those conditions we scored 2 points each. One point was given for age between 65 and 75 years (OR 1.6 [95% CI 1.1–2.5], *P* = 0.03) as well as for use of cardiac glycosides (OR 1.9 [95% CI 1.3–3.4], *P* = 0.003) or loop diuretics (OR 1.5 [95% CI 1.1–2.1], *P* = 0.01). As statin therapy was associated with an OR of 0.5 (95% CI 0.4–0.8, *P* < 0.001), we subtracted 1 point for those patients (Table 3).

**TABLE 2:**
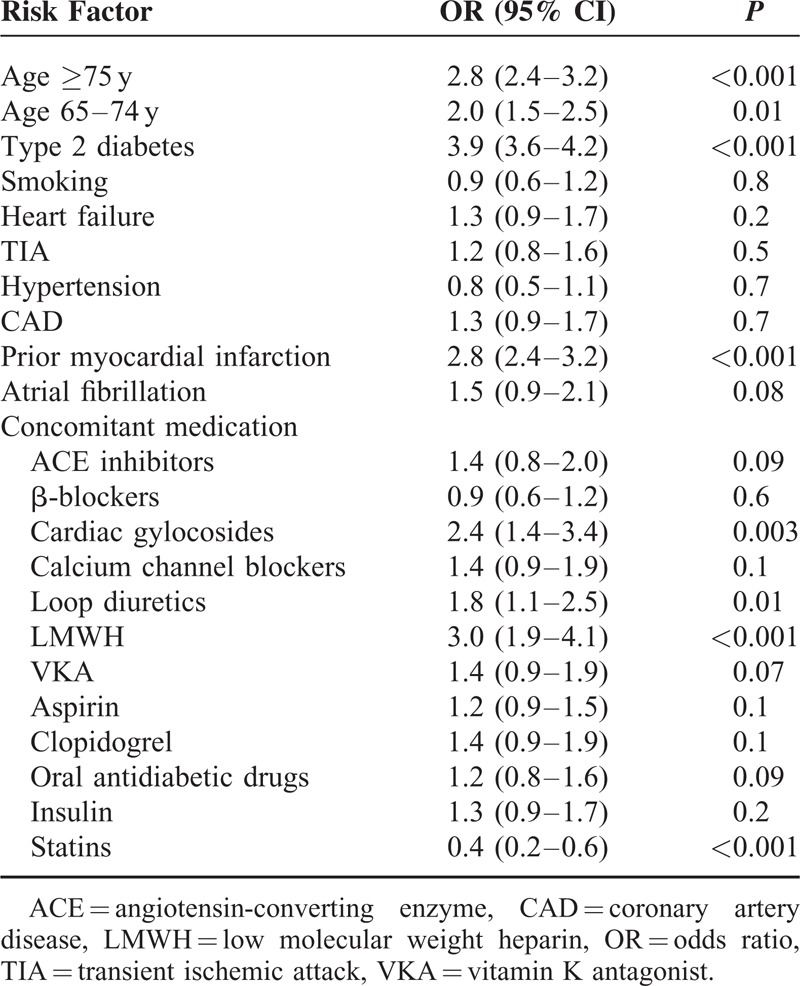
Risk Factors for Critical Limb Ischemia in Peripheral Arterial Occlusive Disease Patients (Cohort 1) in Univariate Regression Analysis

**TABLE 3:**
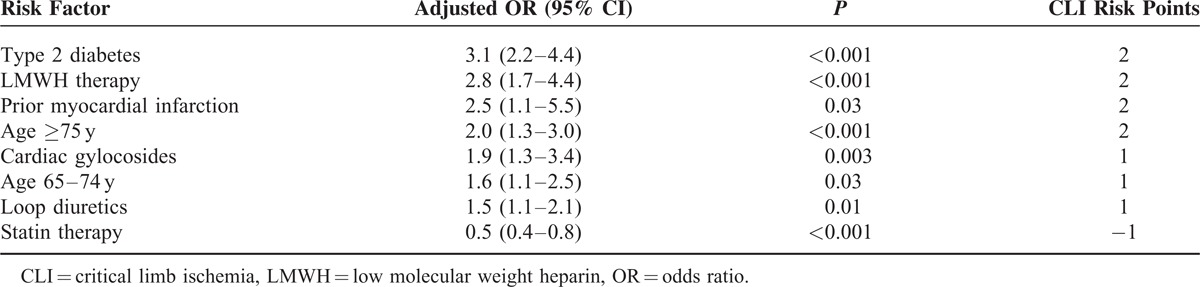
Adjusted Risk Factors for Critical Limb Ischemia in Peripheral Arterial Occlusive Disease Patients (Cohort 1)

**FIGURE 1:**
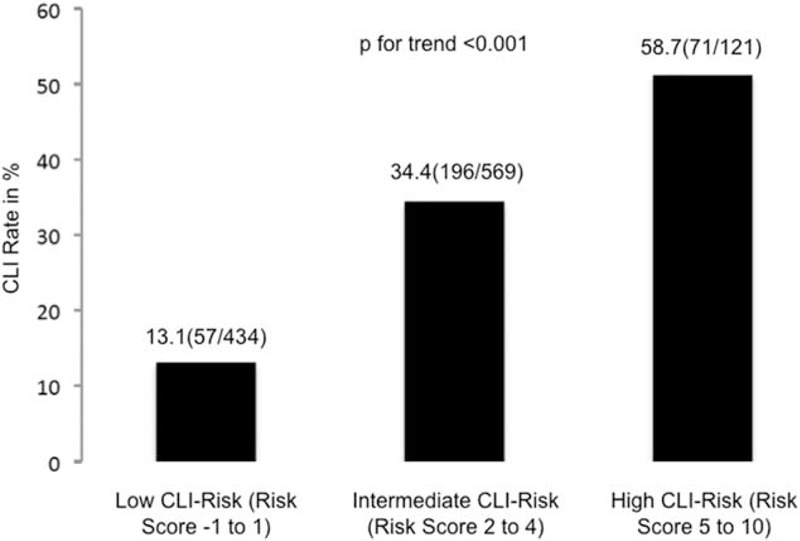
Percentage of patients with critical limb ischemia stratified by critical limb ischemia risk score. Data in the figure are % (CLI patients/all patients in the group).

In the second group of patients, we could prove that the frequency of CLI was significantly higher in patients with a high CLI score (Table 4). The high-risk group (score 6–10) was very small and we actually were not able to show a linear progression for CLI in these patients, but we nonetheless were able to define 3 different risk groups (low CLI risk: score −1 to 1, n = 434; intermediate CLI risk: score 2–4, n = 569; and high CLI risk: score 5–10, n = 121) according to the number of score points (Table 1). The CLI rate was significantly higher in patients with a higher score, especially those with a score of >4 points (Figure 1). The statistical trend we saw for the increase in the CLI rate with increasing score points was statistically significant (*P* < 0.001).

**TABLE 4:**
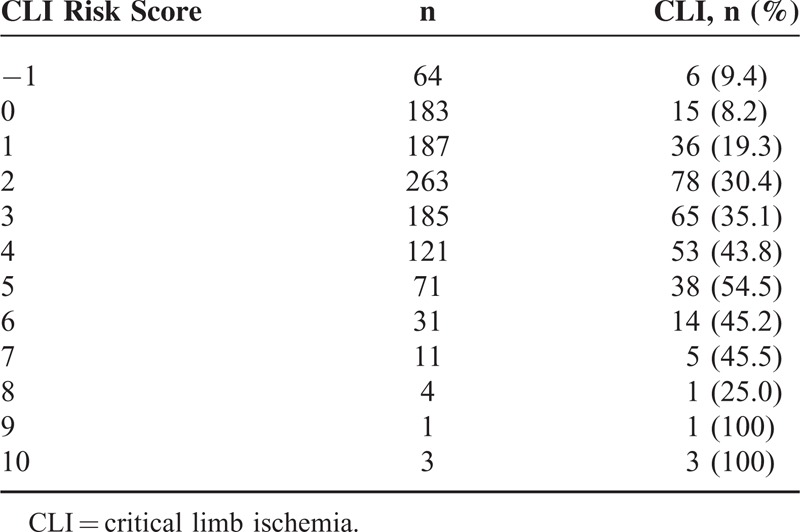
Critical Limb Ischemia Patients According to the Critical Limb Ischemia Risk Score in Cohort 2

We analyzed the correlation of the Graz CLI score with the inflammatory parameters that reflect the activity of the underlying atherosclerotic disease. We calculated Pearson's correlation and Spearman's ρ for the correlation of c-reactive protein (CRP) and fibrinogen with the CLI score and found a highly significant statistical correlation for both parameters (for CRP: Pearson's *r* = 0.3; Spearman's ρ = 0.4; for fibrinogen: Pearson's *r* = 0.2; Spearman's ρ = 0.3; *P* value for all calculations <0.001).

## DISCUSSION

This study demonstrated that the risk for CLI in PAOD patients can be determined easily on the basis of their clinical parameters and concomitant medication. Our score correlates well with inflammatory parameters that reflect the activity of atherosclerosis in these patients.

In general, scoring systems can be useful for identifying patients at high risk for vascular endpoints. The Framingham risk score can be used to highlight patients at high risk for cardiovascular disease^5^ and those with a high-risk score might profit from intense management of their vascular risk factors, as might those with a high Graz CLI score. We hypothesize that those PAOD patients should be treated immediately as their elevated inflammatory parameters suggest a more aggressive atherosclerosis.

With our score, age and diabetes were among the most aggressive risk factors, with an OR of 2.0 and 3.1, respectively. These findings are in accord with similar findings that our group has published recently. We were able to show that an elevated CHA_2_DS_2_-VASc score is associated with an elevated CLI risk.^8^ With this scoring system, diabetes and age >75 years were the 2 main entities associated with CLI.^8^ Both entities are also closely linked to inflammation, especially in atherosclerotic patients. There are signs of chronic inflammation in both, diabetes and insulin resistance, a typical feature of type 2 diabetes.^9^ Inflammation and size of necrotic core are also increased in diabetic patients with CAD.^10,11^ Concerning age there are similar data on age-related inflammation increase in atherosclerotic patients. The reason for this age-related systemic chronic inflammation is mainly attributed to the progressive activation of immune cells over time.^12^

When the Graz CLI score was analyzed, the only protective factor for CLI was concomitant statin medication. Much research has been conducted to evaluate the protective effect of statin therapy, especially in CLI patients. Westin et al^13^ recently showed that statins were associated with lower mortality, a reduction in major adverse cardiovascular and cerebrovascular events, and longer amputation-free survival in CLI patients. Even in patients who have undergone endovascular treatment, statin therapy is associated with superior clinical outcome.^14^ Especially in combination with angiotensin-converting enzyme inhibitors, statins may have even reduced mortality in diabetic CLI patients during a 2-year follow-up.^15^ The beneficial findings of statins in CLI patients were further underlined in large databases, like the REACH Registry.^16^ A recent publication on symptomatic PAOD patients revealed that among them, statin use was associated with an 18% lower rate of adverse limb outcomes, including worsening of symptoms, peripheral revascularization, and ischemic amputations.^16^ Statin therapy should by all means be initiated for all PAOD patients, especially those with diabetes. These recommendations are also reflected in current guidelines, although adherence to them is rather poor.^17^

One further CLI risk factor in our score was glycoside treatment. As this medication is mainly given to patients with congestive heart failure, their low cardiac output causes hemodynamic alterations that include a low flow state, increased peripheral resistance, endothelial cell dysfunction, and vascular smooth muscle cell dysfunction that could exacerbate ischemic symptoms.^18–20^

One further factor associated with CLI in our cohort was a history of MI. We already know that patients with PAOD have a higher risk of fatal and nonfatal cardiovascular events than do patients with symptomatic atherosclerosis in other vascular beds such as the coronary, carotid, and cerebral arteries.^21,22^ The higher values for inflammatory parameters with higher CLI scores might reflect a more active underlying atherosclerosis that in turn could increase their MI rate.

In elderly patients, dehydration often leads to renal impairment and even acute renal failure. Diurectics are often prescribed for the many geriatric patients who suffer from congestive heart failure, and loop diuretics were implicated as a further CLI risk factor in our cohort. Dehydration reduces peripheral perfusion, leading to vascular occlusion and more severe PAOD symptoms, reflected in a higher CLI rate.

Antiplatelet therapy is mandatory for atherosclerosis patients, especially after endovascular treatment, but for some patients, such as those with atrial fibrillation, vitamin K antagonist (VKA) is indicated. When VKA therapy is contraindicated because of a high bleeding risk, LMWH is an alternative. This therapy was a risk factor for CLI in our cohort with an OR of 2.8. One explanation is that these patients might tend to be multimorbid, with highly active underlying atherosclerosis leading to a high CLI rate. There are, however, also data showing that heparins might activate platelets.^23^ This activation of platelets might explain the increase in vascular endpoints including the development of CLI.

The main drawback of our study is its retrospective design. Furthermore, there were statistically significant differences between cohort 1 and cohort 2 concerning incidence of CAD, atrial fibrillation, and comedication (clopidogrel, angiotensin receptor blocker, and β-blocker therapy). As clopidogrel is beneficial especially in PAD patients,^24^ at least some impact on our results cannot be excluded.

With the Graz CLI score, we have developed a simple risk stratification scheme on the basis of information that can be easily acquired from the medical history, without any laboratory parameters. This score proved to be a promising tool to identify PAOD patients at high risk for CLI and should be further evaluated in an upcoming prospective study.
